# A Straightforward Method for 3D Visualization of B Cell Clusters and High Endothelial Venules in Lymph Nodes Highlights Differential Roles of TNFRI and -II

**DOI:** 10.3389/fimmu.2021.699336

**Published:** 2021-06-21

**Authors:** Kim C. M. Jeucken, Jasper J. Koning, Jan Piet van Hamburg, Reina E. Mebius, Sander W. Tas

**Affiliations:** ^1^ Department of Experimental Immunology, Amsterdam Institute for Infection & Immunity, Amsterdam University Medical Center (UMC), University of Amsterdam, Amsterdam, Netherlands; ^2^ Department of Clinical Immunology and Rheumatology, Amsterdam Rheumatology and Immunology Center, Amsterdam University Medical Center, University of Amsterdam, Amsterdam, Netherlands; ^3^ Department of Molecular Cell Biology and Immunology, Amsterdam Infection & Immunity Institute, Amsterdam University Medical Center, Vrije Universiteit Amsterdam, Amsterdam, Netherlands

**Keywords:** whole mount histology, tissue clearing and labeling technique, tumor necrosis factor receptor superfamily, high endothelial venules, peripheral lymph nodes, B cell clusters

## Abstract

Whole mount tissue immunolabeling and imaging of complete organs has tremendous benefits in characterizing organ morphology. Here, we present a straightforward method for immunostaining, clearing and imaging of whole murine peripheral lymph nodes (PLNs) for detailed analysis of their architecture and discuss all procedures in detail in a step-by-step approach. Given the importance of tumor necrosis factor receptor (TNFR) signaling in development of PLNs we used TNFRI^-/-^ and TNFRII^-/-^ mice models as proof-of-concept for this technique by visualizing and analyzing structural changes in PLN B cell clusters and high endothelial venules (HEVs). Samples were subjected to de- and rehydration with methanol, labeled with antibodies for B cells, T cells and high endothelial venules (HEVs) and optically cleared using benzyl alcohol-benzyl benzoate. Imaging was done using LaVision light sheet microscope and analysis with Imaris software. Using these techniques, we confirmed previous findings that TNFRI signaling is essential for formation of individual B cell clusters. In addition, Our data suggest that TNFRII signaling is also to some extent involved in this process as TNFRII-/- PLNs had a B cell cluster morphology reminiscent of TNFRI^-/-^ PLNs. Moreover, visualization and objective quantification of the complete PLN high endothelial vasculature unveiled reduced volume, length and branching points of HEVs in TNFRI^-/-^ PLNs, revealing an earlier unrecognized contribution of TNFRI signaling in HEV morphology. Together, these results underline the potential of whole mount tissue staining and advanced imaging techniques to unravel even subtle changes in lymphoid tissue architecture.

## Introduction

Microscopic visualization of molecular markers and cells with immunolabeling has been among the most important breakthroughs in biological research. For a long time microscopic imaging was constrained to tissue sections with a thickness of mere micrometers, but over the years tremendous advantages have been made accumulating in a plethora of techniques for immunolabeling, optical clearing and imaging of complete organs, animals or even human embryos with minimal disassembly of the tissue (1–5).

In general, these methods apply a combination of whole mount immunofluorescence (IF) staining, optical tissue clearing and ultramicroscopy (UM) imaging (1). Using state-of-the-art software, raw data can be processed into 3 dimensional (3D) reconstructions of the original tissue that can be used for detailed analyses. Together, these techniques provide a big step forward in the field of histological research. However, not all methods are suited for every tissue type and adaptations are dictated by the specific composition of the tissue of interest. For instance, organs consisting of high amounts of connective tissue have proven to be relatively difficult to clear (6). Fortunately, also methods exist – in particular those using solvent-based clearing - that can successfully be applied to many tissues and whole organs (5, 7–12). These organs include lymph nodes (LNs), which are relatively easy to subject to techniques for 3D visualization and analysis.

LNs are distributed throughout the body and form central sites for initiation and shaping of immune responses by facilitating interactions between incoming (naïve) immune cells and local antigen presenting and stromal cells (13). To efficiently enable these interactions, LNs have an organized architecture which includes several main elements such as individual B cell follicles located in the cortex, a central T cell zone in the paracortex and a vascular network consisting of lymphatic and blood vessels (13). The latter contains the specialized high endothelial venules (HEVs) that mediate in extravasation of (naïve) lymphocytes (14). Over the years, much research has been done on LN morphology, development and function, leading to a broad understanding of the cells and signaling pathways involved. Among the key players required for development of fully functional LNs are the TNF receptor superfamily (TNFRSF) members (15, 16).

The TNFRSF consists of many different receptors, several of which are critically important in the context of LN development; i.e., mice lacking lymphotoxin β receptor (LTβ)R (TNFRSF3) or receptor activator of nuclear factor κB (RANK/TNFRSF11A) fail to develop functional LNs (17, 18). In addition, also TNFRI signaling is involved in LN development. Although mice lacking TNFRI signaling have functional LNs, they lack clear individual B cell clusters (19, 20). Furthermore, it has been proposed that TNFRI signaling plays a role in development of HEVs (14, 21). The role of TNFRI signaling in B cell follicle architecture was unraveled using straightforward histological techniques (i.e. hematoxylin and eosin (HE) staining), but proving a role of TNFRI signaling in HEV formation is more challenging given its complex 3D structure (19, 20, 22). For instance, using confocal microscopy tissue thickness is highly limited, making it impossible to quantify intricate features like total vasculature volume and length. So, since not all HEV characteristics can be investigated using tissue slices, it is hard to prove or dismiss a definitive role for TNFRI signaling. Therefore, whole-mount LN analysis might uncover currently unknown roles for TNFRI.

Another well-known TNFRSF member that is currently deemed dispensable for LN development is TNFRII. Despite the fact that ligands for TNFRI and TNFRII signaling (i.e. TNFα, LTα) largely overlap, no role for TNFRII signaling in LN development has been described as of yet (23). Nevertheless, it is possible that a more subtle role for TNFRII in LN morphology has been missed due to a lack of appropriate analytic methods.

Here, we provide a detailed description of a straightforward method for whole organ immunostaining, clearing, imaging and morphological analysis of both robust and complex structures in LNs. To illustrate the potential of this method, we analyzed morphological changes in B cell cluster and HEV morphology in peripheral (P)LNs of TNFRI^-/-^ and TNFRII^-/-^ mice.

## Material and Methods

### Mice

TNFRI^−/−^ (Tnfrsf1a^tm1Imx^) and TNFRII^−/−^ (Tnfrsf1b^tm1Mwm^) mice were obtained from The Jackson Laboratory. TNFRI^−/−^, TNFRII^−/−^ and WT littermates on C57Bl/6 background (C57Bl/6JOlaHsD; Harlan, The Netherlands) were bred and housed in specific pathogen-free conditions in the Academic Medical Center animal facility. Experiments were performed in accordance with national legislation and under supervision of the Animal Experimental Committee of the Academic Medical Center and the University of Amsterdam.

### Sample Preparation

**Figure 1:**
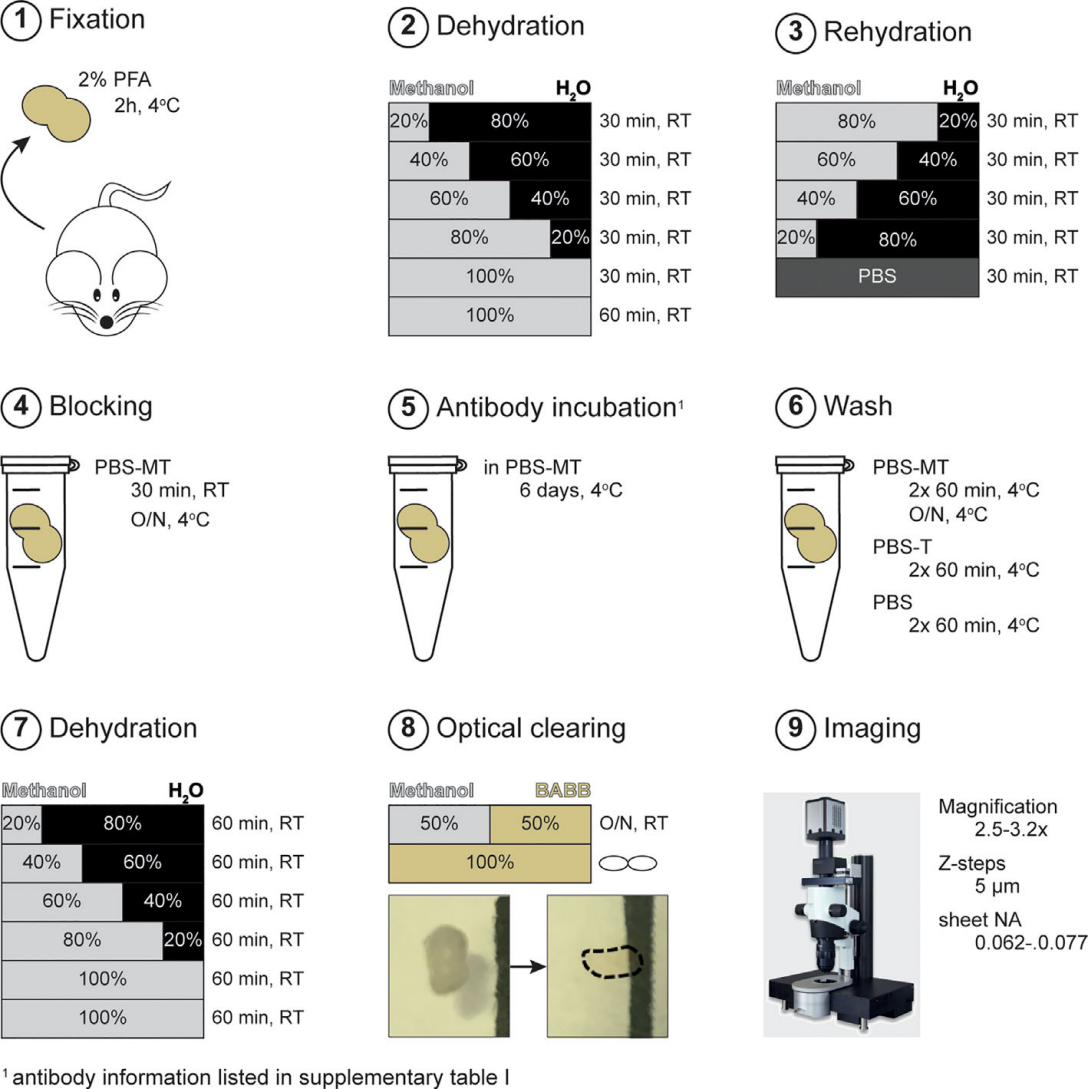
Schematic representation of experimental procedure. After fixation (step 1), samples were subjected to de- and rehydration using methanol/H_2_O series (step 2-3). Next, samples were overnight blocked with PBS-MT followed by antibody incubation (step 4-6). Sample clearing was achieved by methanol dehydration and incubation with BABB solution (step 7-8). Sample imaging was done using ultramicroscopy (step 9). PBS-MT; 1% skim-milk + 0.4% TritonX-100 in PBS. RT, room temperature; O/N, overnight.

PLNs (brachial and inguinal) were isolated using fine forceps to keep the tissue intact and collected in ice-cold PBS until further handling. After, removal of excess fat tissue, samples were fixed for 2h in 2% methanol-free formaldehyde (Thermo Scientific, Waltham) in PBS at 4°C while rolling, washed 3x 30 minutes with cold PBS and stored in 0.02% NaN_3_/PBS at 4°C until further processing (**Figure 1**, step 1; **Supplementary Table 1**).

### Dehydration and Rehydration

To ensure optimal antibody labeling and clearing of tissues, liquids and lipids were removed by methanol dehydration and the subsequent replacement of H_2_O *via* rehydration series. Dehydration was done in the following order; 20%, 40%, 60%, 80%, 100% (v/v; methanol/H_2_O), all 30 minutes, followed by a final 100% dehydration step for 1h (**Figure 1**, step 2). Next, samples were rehydrated in reverse order; 80%, 60%, 40%, 20%, all 30 min Lastly, samples were washed with PBS for 30 minutes (**Figure 1**, step 3). Dehydration, rehydration and washing steps were all performed at room temperature (RT) under continuous rotation (**Supplementary Table 1**). Samples were kept in glass containers (VWR, Radnor) during de- and rehydration steps to prevent fluid-leakage due to methanol-induced corrosion of plastic tubes (**Supplementary Table 1**).

### Immunofluorescent Labeling

To prevent a specific anti-body binding and to minimize background staining, samples were blocked with PBS-MT (1% skim-milk (Biorad, Hercules), 0.4% TritonX-100 (Sigma-Aldrich, St Louis)); 30 min RT and overnight, 4°C (**Figure 1**, step 4). The next day, PLN were incubated directly-labeled antibodies against CD3 (AlexaFluor (AF) 488; Biolegend, San Diego), B220 (AF594; Biolegend) and MECA-79/PNAd (DyLight (DL) 633; gift from E. Butcher lab, Stanford) in PBS-MT for 6-7 days at 4°C, in the dark (**Supplementary Table 1**) (**Figure 1**, step 5). After immunofluorescent labeling, samples were extensively washed with PBS-MT 2x 60min, and 1x overnight at 4°C; PBS-T 2x 60 min at 4°C followed by several rinses; and PBS 2x 60 min at 4°C (**Figure 1**, step 6). Samples were saved in PBS at 4°C until clearing. All blocking, immunofluorescent labeling and washing steps were done under continues rotation.

### Optical Clearing

For better handling of the tissue, samples were embedded in 1.5% low melting Ultrapure agarose (Thermo Scientific) in H_2_O prior to clearing. To remove all liquids, samples were again subjected to dehydration with methanol; 20%, 40%, 60%, 80%, 100% (v/v; methanol/H_2_O), all 60 minutes. Next, a final dehydration step was performed with 100% methanol to ensure complete removal of water (**Figure 1**, step 7). After dehydration, samples were overnight incubated with a 1:1 (v/v) solution of methanol: BABB (Benzyl Alcohol (Sigma-Aldrich)/Benzyl Benzoate (Sigma-Aldrich); 1:2 (v/v)). Next day, the methanol: BABB solution was replaced with 100% BABB (**Figure 1**, step 8). In general, full optical clearing was achieved within 3 h. When PLNs were not fully cleared within 8h, BABB solution was refreshed. Fully cleared PLNs were stored in BABB solution at RT in the dark until imaging. Dehydration and methanol: BABB incubation were performed at RT, under continuous rotation and in the dark. Importantly, presence of air bubbles within sample tubes was avoided at all times to prevent oxidation that could possibly interfere with complete dehydration and optical clearing. Of note, clearing procedures were performed in glass containers (**Supplementary Table 1**).

### Ultramicroscopy Imaging

Imaging of optically cleared PLNs was done by light sheet microscopy (LSM) using the LaVision UltraMicroscope II (Miltenyi, Bielefeld) with the following specifications: bidirectional illumination with a total of 6 light sheets, sheet width range of 1-20 mm, sheet numerical aperture (NA) of 0.0135-0.135, 0.5 NA objective lens, and equipped with a super continuum laser (emission range 460-800 nm) and the following filter sets; 470(40)/525(50); 545(30)/595(40); 630(30)/700(75); 785(25)/845(55) (excitation/emission)). Microscopy set-up and imaging acquisition was performed as previously described by Erturk et al. (5). In this study, sample acquisition was done with a step size of 5 μm and a sheet NA of 0.062-0.077. The used magnification was 2.5 or 3.2x, depending on the highest magnification possible for visualization of the entire sample (**Figure 1**, step 9).

### Data Analysis

**Figure 2:**
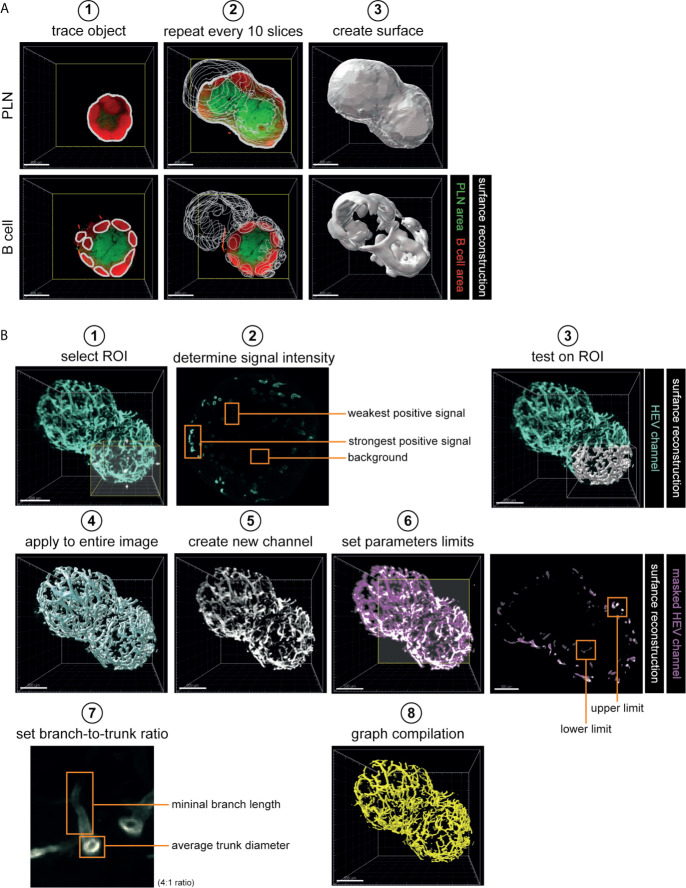
Analytical work-flow. **(A)** 3D reconstruction of whole PLN and B cell cluster morphology. 3D volumetric surfaces were created using Imaris Surface tool, using Manual mode. Manual tracking of every 10 slices which corresponded with 50 µm sample thickness (step 1-2). The acquired surface reconstructions were used for analysis of morphology and volume of whole PLNs and B cell clusters (step 3). **(B)** 3D reconstruction and analysis of high endothelial vasculature. 3D volumetric surfaces were created using Imaris Surface tool, using Threshold mode (step 1-5). For each individual sample, threshold parameters were tested and set based on weakest positive signal (step 1-4). The acquired 3D surface was used to create a masked HEV channel (step 5). Masked HEV channel was used to extract data on HEV morphology by Imaris Filament Tracer tool using the Threshold (loops) mode (step 6-8). Algorithm parameters were set based on lower and upper limit of signal threshold (step 6). To exclude inference of insignificant protrusions in branching point analysis a branch-to-trunk ratio of 4:1 was used (step 7). Data acquired by Surface tool was used for analysis of vessel volume (step 4). Data acquired by Filament Tracer Tool was used for analysis of vessel length and branching points (step 8). Scale bars: 500 μm (**A, B**; step 1) and 300 μm (**B**; step 3-8). PLN, peripheral lymph node; ROI, region of interest; HEV, high endothelial venule.

Data analysis was performed using Imaris software (version 9.5.1; Oxford instruments, Belfast). First, 3D volumetric surface were generated. To generate surface reconstructions of whole PLNs (based on autofluoresence), B cell clusters (based on B220 staining) and T cell areas (based on CD3) the manual tracking feature of Imaris Surface tool was used. To reconstruct 3D volumes, sample outline (PLN volume) or outlines of B220^+^ (B cell clusters) or CD3^+^ (T cell areas) areas were traced (for every 10 slides = 50 µm) (**Figure 2A**). Next, a surface reconstruction was automatically calculated which was used for further analysis (**Figure 2A**). Of note, for B cell cluster analysis only objects > 1.0*10^5^ µm^3^ were considered as true clusters and included.

For volumetric and morphological analysis of high endothelial vasculature, Imaris Surface and Imaris Filament Tracer tools were used. A 3D volumetric surface of the vasculature was generated using the Threshold feature of Imaris Surface tool. Threshold values used to generate 3D reconstructions were determined based on background signal and intensity of the weakest positive signal of the HEV channel. These parameters were then tested on a region of interest (ROI) - and optimized if necessary - before being applied to the entire sample to generate a surface reconstruction which was then used for analysis of HEV volume. Next, the surface reconstruction was used to create a new HEV channel that completely lacked background signal (masked HEV channel) and which could be used for further analysis (**Figure 2B** steps 1-5, **Supplementary Figure 1A**). To quantify HEV length and branching points, we used Imaris Filament Tracer tool with Threshold (loops) mode. First, algorithm parameters were set by determining the lower and upper limits of the signal intensity to be included in calculations. Next, we set parameters for vessel bifurcations; in this study we classified sprouting vessels as vessels that had a length of at least 4 times the diameter of the main vessel (4:1 branch: trunk ratio). This latter avoided contamination of insignificant protrusions when calculating the amount of branching points (**Figure 2B** steps 6-8, **Supplementary Figure 1B**). Of note, since small differences in signal intensity between samples are inevitable, threshold parameters were defined for each individual sample. To avoid bias, samples were analyzed in random order.

### Statistics

For all analyses, significance was determined using Student’s two-tailed unpaired t-test using GraphPad Prism v8.2.1 (Graphpad Software Inc.). Differences were considered significant for p values ≤ 0.05 (* = p ≤ 0.05). For all analyses *n* = 6 for WT and TNFRI^-/-^, and *n* = 5 for TNFRII^-/-^. Data is shown as mean and SEM.

## Results

### Visualization of Total PLN Volume and T Cell Areas in WT, TNFRI^-/-^, and TNFRII^-/-^ Mice

To illustrate the application of the above describe method for the analysis of potential differences in robust structures within PLNs, we compared PLNs of WT mice with PLNs of mice deficient in either TNFRI (TNFRI^-/-^) or TNFRII (TNFRII^-/-^). Both TNFRI^-/-^ and TNFRII^-/-^ mice developed PLNs that were macroscopically similar to WT. In addition, 3D reconstruction did not indicate differences in PLN surface morphology (**Supplementary Figure 2**). Similar, no visual differences in T cell areas were observed between the different phenotypes (**Supplementary Figure 2**). In addition, total PLN volume calculated based on 3D volumetric surface was similar for all genotypes (TNFRI^-/-^; 2.23 ± 0.311 mm^3^, or TNFRII^-/-^; 2.17 ± 0.401, WT; 2.18 ± 0.422) (data not shown).

### 3D Reconstruction Confirms Role for TNFRI Signaling in Formation of Individual B Cell Clusters in PLNs

Next, we visualized and quantified potential changes in B cell clusters of TNFRI^-/-^ and TNFRII^-/-^ PLNs compared to WT mice.

**Figure 3:**
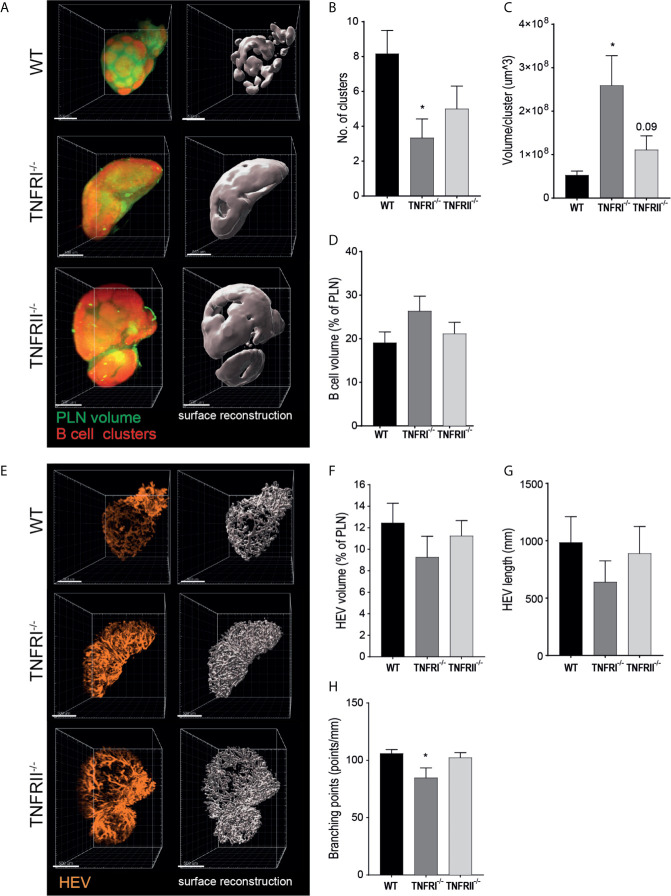
TNFR signaling is involved in B cell cluster and HEV morphology. TNFRI- and TNFRII-signaling are involved in formation of individual B cell clusters **(A–D)**. **(A)** representative UM image (left; total PLN volume in green, B cell clusters in red) and 3D volumetric reconstruction (right; B cell clusters shown in white) of PLN for each phenotype. **(B)** number of individual B cell clusters per PLN. **(C)** average volume per B cell cluster. **(D)** total B cell volume. TNFRI-, but not TNFRII-deficiency affects HEV morphological features **(E–H)**. **(E)** representative UM image (left) and 3D volumetric reconstruction (right) of high endothelial vasculature for each phenotype. **(F)** total HEV volume. **(G)** total HEV length. **(H)** number of branching points. Scale bars: 500 μm. PLN; peripheral lymph node. *n = 5* to *6.* *p < 0.05.

Previous studies using traditional HE or IF staining already demonstrated that TNFRI signaling is critical for correct organization of B cell follicles (19, 20). Visualization of the complete PLN B cell architecture confirmed this earlier findings that TNFRI^-/-^ mice lack individual B cell clusters, but rather have a single peripheral sheet of B cells at the PLN perimeter (**Figure 3A**, **Supplementary Figure 2**). Unexpectedly, B cell clusters of TNFRII^-/-^ PLNs also appeared less well defined although the visual differences between TNFRII^-/-^ and WT PLNs were far less apparent than those observed in TNFRI^-/-^ PLNs (**Figure 3A**). To quantify the observed differences in B cell cluster morphology, we calculated the number and volume of individual B cell clusters. As expected, the number of B cell clusters in TNFRI^-/-^ PLNs was decreased (**Figure 3B**), while the volume per cluster was strongly increased (**Figure 3C**). Of note, B cell clusters in TNFRII^-/-^ PLNs also appeared to be decreased although to a much lesser extent than observed in TNFRI^-/-^ PLNs (**Figures 3B, C**).

To check whether morphological changes affected overall B cell volume, we quantified total B cell volume. No significant changes were detected in either TNFRI^-/-^ or TNFRII^-/-^ PLNs (**Figure 3D**). In addition, similar data about total B cell numbers was obtained using FACS analysis (data not shown).

These data highlight the potential of 3D UM for morphological analysis of robust structures like PLN B cell clusters. Moreover, we found subtle changes in TNFRII^-/-^ PLNs that had not been established previously by traditional histology.

### 3D Reconstruction and Analysis of High Endothelial Vasculature in PLNs Suggests a Previously Unknown Role for TNFRI

Whereas analysis of B cell clusters using tissue slices is possible to some extent, this is far more challenging for analysis of HEVs. Given the complexity of the HEV network (i.e. vessel branching, winding of vessels) there is a risk of missing structural alterations when using thin tissue sections for analysis. To overcome these hurdles, we presented here a workflow that allowed analysis of the different features of the complex HEV network (**Figure 2B**). As a result, we were able to not only visualize the complete high endothelial vasculature (**Figure 3E**, **Supplementary Figure 2**) but also objectively quantify differences in volume, length and branching points to check for differences between WT, TNFRI^-/-^ and TNFRII^-/-^ PLNs.

We observed a trend towards a decrease in HEV volume in TNFRI^-/-^ PLNs (~25% decrease), whereas only slight differences were found in TNFRII^-/-^ PLNs (~10%) (**Figure 3F**). In addition, similar data for HEV volume was obtained using FACS; a slight decrease in high endothelial cells in TFNRI^-/-^ and no change in TFNRII^-/-^ PLNs (% of blood endothelial cells; data not shown). Similar to HEV volume, we observed a trend towards decreased total HEV length in TNFRI^-/-^ (~35% decrease) and only minimal changes in TNFRII^-/-^ animals (~10%) (**Figure 3G**). Lastly, we quantified the extent of HEV branching points and found a significant decrease in branching of HEVs in TNFRI^-/-^PLNs, whereas no differences were found in TNFRII^-/-^ PLNs (**Figure 3H**).

Taken together, using UM and 3D reconstruction of the entire HEV network we were able to objectively quantify different HEV characteristics and suggest a role for TNFRI signaling in HEV volume, length and branching in PLNs.

## Discussion

In the current study we described a straightforward method for whole mount tissue IF staining, optical clearing, UM imaging and analysis of PLN morphology. To illustrate the potential of this method to visualize and analyze both robust and complex structures, we quantified morphological changes in PLN B cell clusters and HEVs of TNFRI^-/-^ and TNFRII^-/-^ mice. First, we confirmed the known role for TNFRI signaling in formation of individual B cell clusters and additionally our data suggest that a possible role for TNFRII signaling in B cell architecture since TNFRII^-/-^ PLN B cell follicles exhibited a morphology that mildly resembled those of TNFRI^-/-^ mice (19, 20). Moreover, our data suggest a role for TNFRI signaling in different aspects of HEV morphology, like vessel length and branching. Together, this underlines the value of whole organ analysis to identify morphological changes, especially in the context of subtle changes that are likely to be missed when analyzing thin tissue slices.

Nowadays many methods for whole organ labeling and imaging exist, which differ mostly in optical clearing procedures. These procedures can roughly be divided into tissue transforming, hydrophilic, and solvent-based methods (24, 25). Solvent-based clearing methods - which we have used here - use organic solvents such as dibenzyl ether and BABB to render tissues transparent (24, 25). As with the method described here, most solvent-based clearing procedures are relatively straight-forward and can easily be used for different tissue types (25). The biggest advantages of solvent-based clearing procedures are that they 1) are fast, 2) obtain a great level of transparency and 3) that cleared samples can be stored for a long time (6, 25). However, there are also disadvantages; for instance, the harsh chemical treatments may cause isotope and/or fluorescence signal loss and can lead to tissue shrinkage (6, 24, 25).

While tissue shrinkage may have technical advantages in some cases – i.e. shrinking of large tissue samples reduces imaging time - it is a significant disadvantage when measuring biological quantities like volume and size. This is especially troubling when performing side-by-side comparisons of studies that used different clearing methods. The methanol dehydration and BABB clearing we used here are known to induce significant tissue shrinking (3, 6). Although we did not determine the exact amount of shrinking here, we are aware that the volumes and sizes reported are likely to be an underestimation of the real biological values. Previous studies report a shrinkage of up to 50% of BABB treated tissues and it is likely that the amount of shrinkage in our study is similar, meaning that biological values might be up to 2 times higher (24, 26). However, we expect tissue shrinkage to be equal for all samples, so it should not affect the observed differences between WT, TNFRI^-/-^ and TNFRII^-/-^ PLNs.

The method described here is suited for the analysis of structural components that can easily be identified by a limited amount of markers (i.e. B220 for B cell clusters and MECA-79 for HEVs). Due to technical aspects of most UM equipment however, application of our method remains restricted to a few markers (for the equipment used here; 4 filter sets, possibility to extend to 7). As a consequence, identification of cellular subsets that require multiple markers is challenging. Therefore, our method might be best applied for studying of large structural components. However, other studies have administered multi-color acquisition with UM to reveal heterogeneity at the single cell level (12). Alternatively, other methods like histo-cytometry may better be used to identify organization of specific (single) cells or markers (27). In addition, current techniques for antibody stripping and re-probing on formalin-fixed paraffin-embedded sections offer promising perspectives if these can also be applied to UM samples (28).

Visualization and analysis of vasculature networks by UM methods, including analysis of the LN HEV network, has been done previously (2, 3, 9–12, 22). Although using similar methods to quantify HEV length and branching, these studies measured values ten times lower than we report here (9–11, 22). These differences are likely to arise from differences in immunostaining, imaging modalities or variations in analysis methods. One obvious difference in methodology is the mode of tissue labeling; whereas we performed immunolabeling *ex vivo* after LN harvest and fixation, other studies labeled HEV *via in vivo* intravenous (IV) antibody injection prior to animal sacrifice and LN excision (9–11, 22). The shorter incubation time during *in vivo* labeling (15-20 min *vs* 6-7 days with our method) might be insufficient for antibody penetration through the entire HEV network, especially the smallest vessels. Moreover, HEVs can express PNAd (MECA-79) both at the luminal and abluminal surface. IV antibody injection exclusively labels luminal PNAd, whereas *ex vivo* immunostaining might also labels abluminal PNAd, resulting in different HEV measurements if these are based solely on MECA-79 expression. The use of different labeling techniques may, at least partly, explain the differences in HEV length and branching. This will be fairly easy to test in future experiments *via* IV injection of MECA-79 antibody in one fluorophore followed by *ex vivo* MECA-79 labeling with another fluorophore and subsequently analyzing overlap and differences between the two fluorescence signals. In addition to variation in labeling method, the studies by Chai et al. and Kumar et al. used optical projection tomography (OPT) for imaging whereas we used LSM (9, 10, 22). In general, the resolution of OPT is lower compared to LSM and as a consequence, smaller vessels might be missed with OPT leading to underestimation of the total HEV network (29).

The importance of TNFRI signaling in the development of individual B cell follicles has long been established (19, 20). It is thought that TNF-TNFRI mediated interactions between B cells and follicular dendritic cell (FDCs) are essential in establishing a FDC network. As a consequence, loss of TNFRI signaling and subsequent absence of FDCs affects formation of individual B cell follicles (19, 30–32). We confirmed this by showing that TNFRI^-/-^ PLN lack separate B cell clusters, but rather have one large peripheral B cell sheet. Additionally, we found that TNFRII^-/-^ PLN B cell clusters had a phenotype that to some extent resembled that of TNFRI^-/-^ PLNs. Although the effects we observed were only very mild and further studies are warranted to prove or dismiss a role for TNFRII signaling in PLN B cell morphology, our data suggest that there might be an as-of-yet unknown role for TNFRII in this process as well. These possible alterations might be attributed to defective TNFRII signaling in T cells since TNFRII ligation is associated with activation and differentiation of the T cells that provide B cell help and/or control B cell responses within PLN follicles (33–35). Thus, defective TNFRII signaling in T cells might affect B cell responses, and subsequently B cell follicle morphology. Alternatively, TNFRII loss might also affect FDC function since there substantial overlap between TNFRI and TNFRII signaling in for example DC survival, and as such this may also affect B cell clustering (36).

Previously, it has been proposed that TNFRI signaling is involved in the generation of immature HEVs, although blocking TNFRI signaling does not affect development of mature HEVs (14, 15). In line with this, we did not find visual differences in HEVs of TNFRI^-/-^ PLNs. However, objective quantification of the HEV network did establish a role for TNFRI signaling in HEV morphology as we found that TNFRI^-/-^ PLNs have a slight decrease in HEV volume, length and number of branching points. Consequently, although TNFRI signaling is not essential for HEV development, it might be important for establishing a complete and intricate HEV network. However, future studies are needed to determine the exact role of TNFR signaling in PLN HEVs. Here it is of interest that LTα, which signals *via* TNFRI, induced PNAd expression exclusively at the basolateral surfaces of pancreatic blood vessels. Therefore it would be of interest to investigate whether TNFRI signaling has specific effects in abluminal versus luminal HEV markers (37). To exclude that changes in the HEV compartment are due to alterations in other LN compartments - like B cell clusters – studies with HEV-specific TNFRI^-/-^ animal models are preferred here.

In conclusion, our study highlights the potential of a straightforward method for 3D imaging to study changes in organ morphology. As proof-of-concept we demonstrated both known roles for TNFR signaling in PLN architecture and additionally showed that novel, more subtle, roles can be identified using the described method.

## Data Availability Statement

The original contributions presented in the study are included in the article/**Supplementary Material**. Further inquiries can be directed to the corresponding author.

## Ethics Statement

The animal study was reviewed and approved by Animal Experimental Committee of the Academic Medical Center and the University of Amsterdam.

## Author Contributions

Conceived and performed the methodology and experiments: KJ and JK. Analyzed the data: KJ. Wrote the original draft: KJ. Writing, review and editing: KJ, JK, JH, RM, and ST. All authors contributed to the article and approved the submitted version.

## Funding

This work was supported by the Dutch Arthritis Foundation grant to ST (RF16-1-302) and the Netherlands Organization for Scientific Research grant to JK (ALWOP.271).

## Conflict of Interest

The authors declare that the research was conducted in the absence of any commercial or financial relationships that could be construed as a potential conflict of interest.

## Supplementary Material

Supplementary Figure 1 Representative parameters used for HEV surface reconstruction and quantification. **(A)** Parameters Surface Tool, Threshold mode. **(B)** Parameters Filament Tracer Tool, Threshold (loops) mode.

Supplementary Figure 2 Representative images of 3D reconstructions. **(A)** Unprocessed whole lymph node CD3, B220 and MECA-79 data **(B)** Processed 3D reconstructions of T cell areas, B cell clusters and HEV shown in different combinations. Scale bars: 500 µm.
