# Supplementary figures and images for: A Straightforward Method for 3D Visualization of B Cell Clusters and High Endothelial Venules in Lymph Nodes Highlights Differential Roles of TNFRI and -II
Source: Front Immunol. 2021 Jun 21;12:699336. doi: 10.3389/fimmu.2021.699336 (PMC8255985; doi:10.3389/fimmu.2021.699336)

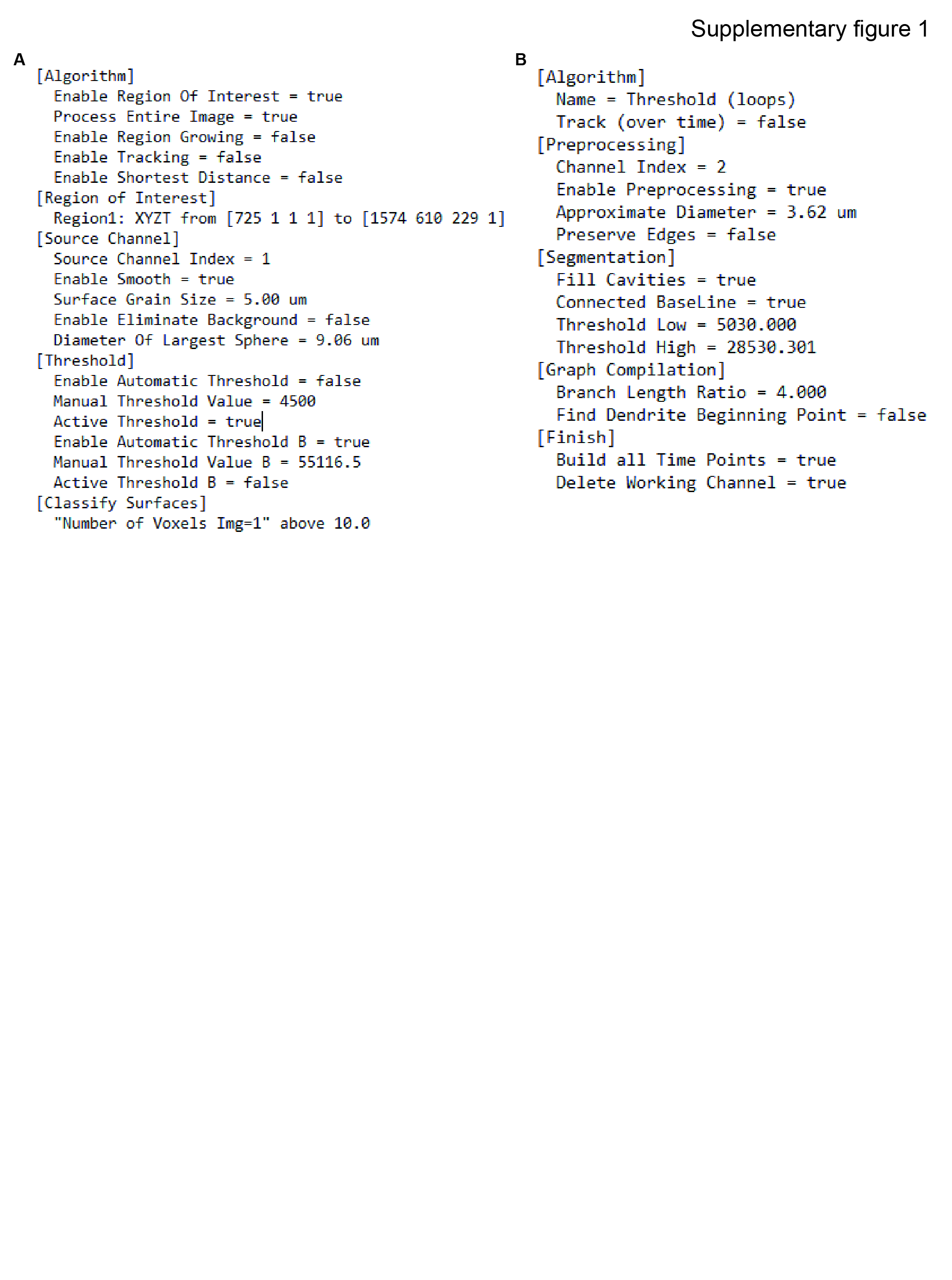

Supplement: Supplementary Figure 1 — Representative parameters used for HEV surface reconstruction and quantification. (A) Parameters Surface Tool, Threshold mode. (B) Parameters Filament Tracer Tool, Threshold (loops) mode. [file Image_1.tif]

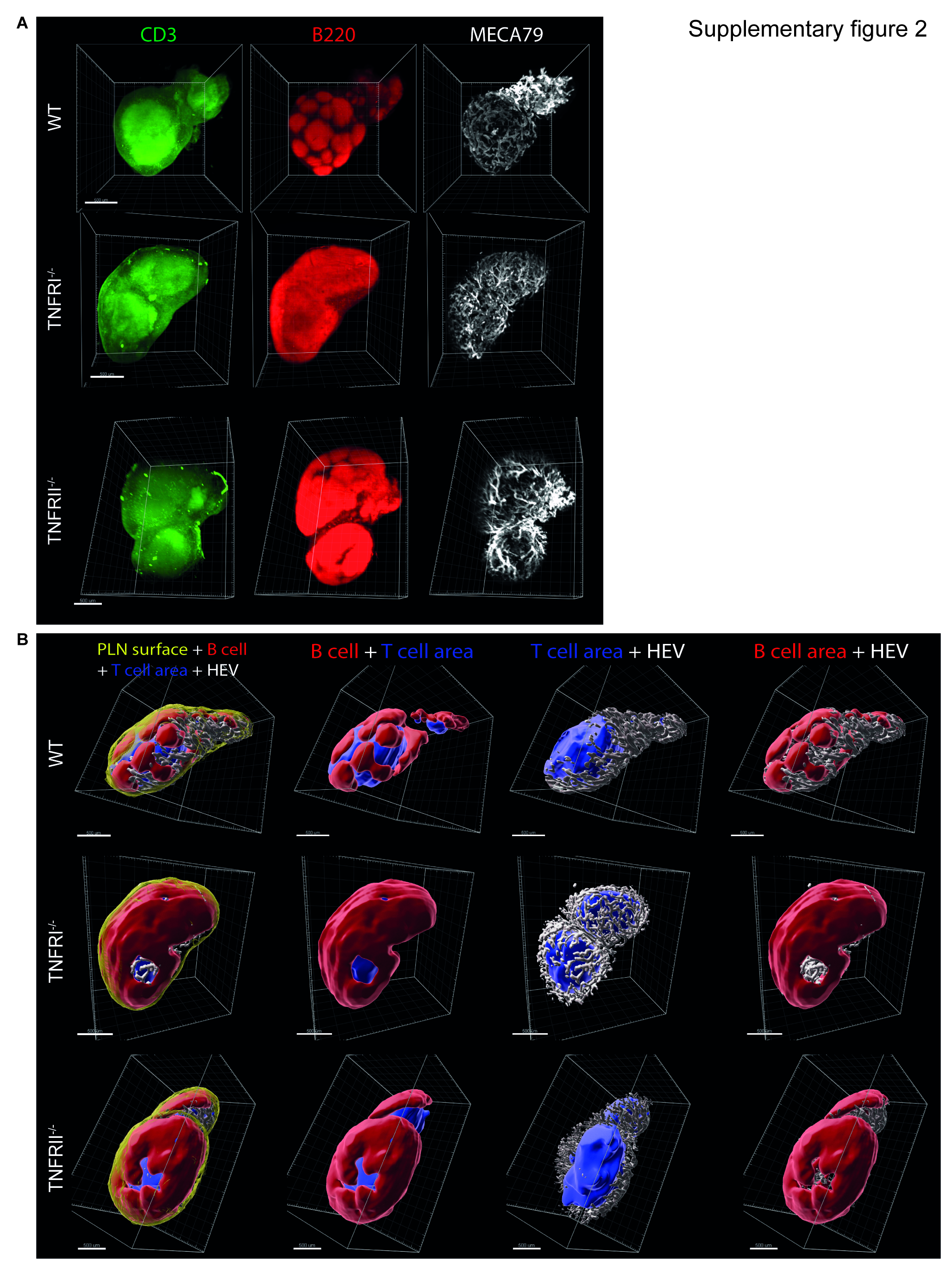

Supplement: Supplementary Figure 2 — Representative images of 3D reconstructions. (A) Unprocessed whole lymph node CD3, B220 and MECA-79 data (B) Processed 3D reconstructions of T cell areas, B cell clusters and HEV shown in different combinations. Scale bars: 500 µm. [file Image_2.tif]
