# Supplementary material for: A Straightforward Method for 3D Visualization of B Cell Clusters and High Endothelial Venules in Lymph Nodes Highlights Differential Roles of TNFRI and -II
Source: Front Immunol. 2021 Jun 21;12:699336. doi: 10.3389/fimmu.2021.699336 (PMC8255985; doi:10.3389/fimmu.2021.699336)
Supplement: Supplementary file 3 [file Table_1.pdf]

Supplementary table 1

Reagent	Volume/sample	Container
4% PFA fixation	1.5 ml	1.5 ml plastic tube
Methanol/H2O series	10 ml	10 ml glass container
PBS-MT (blocking)	500 ul	0.5 ml plastic tube
Antibody mix	500 ul	0.5 ml plastic tube
PBS-MT, PBS-T, PBS (wash)	500 ul	0.5 ml plastic tube
Clearing solution	10 ml	10 ml glass container

Antibody	Label	Host and Isotype	Clone	Manufacturer	Catalog no	Dilution	Concentration
anti-mouse CD3	AF488	Rat IgG2b	17a2	Biolegend	1101060	1:200	2.5 ug/ml
anti-mouse B220	AF594	Rat IgG2a	RA3-6B2	Biolegend	103254	1:200	2.5 ug/ml
anti-mouse MECA-79/PNAd	DL633	Rat IgM	NA	E. Butcher lab. Stanford	NA	1:400	5 ug/ml

Supplementary Table 1 List of used reagents
